# The complete chloroplast genome sequence of *Xylosma racemosa* (Siebold & Zucc.) Miq. (Salicaceae)

**DOI:** 10.1080/23802359.2021.1975509

**Published:** 2021-09-17

**Authors:** Lili Tong, Lu Tian, Xiaogang Xu, Chongli Xia, Yao Cheng, Xiaoyu Jiang, Hongchao Wang, Yukun Tian

**Affiliations:** a School of Horticulture & Landscape Architecture, Jinling Institute of Technology, Nanjing, China; b Co-Innovation Center for Sustainable Forestry in Southern China, College of Biology and the Environment, Key Laboratory of State Forestry and Grassland Administration on Subtropical Forest Biodiversity Conservation, Nanjing Forestry University, Nanjing, China; c State Environmental Protection Scientific Observation and Research Station for Ecology and Environment of Wuyi Mountains, Nanping, China

**Keywords:** *Xylosma racemosa*, phylogenomics, Salicaceae, complete chloroplast genome

## Abstract

*Xylosma racemosa* (Siebold & Zucc.) Miq. which plays an important role in ecology, medicine and economy, is a deciduous species of Salicaceae. In this paper, we sequenced, assembled, and annotated the chloroplast (cp) genome of *X*. *racemosa* (Siebold & Zucc.) Miq. by using the sequencing data from Illumina Novaseq platform. The complete cp genome of *X. racemosa* is 157,262 bp in length, containing a large single-copy (LSC) region of 84,289 bp, and a small single-copy (SSC) region of 17,817 bp. It contains 131 genes, including 8 rRNA genes, 37 tRNAs genes and 86 protein-coding genes. The GC content of *X. racemosa* cp genome is 36.74%. The phylogenetic analysis suggests that *X. racemosa* is a sister species to *Xylosma congesta* in Salicaceae.

*Xylosma racemosa* (Siebold & Zucc.) Miq. (Miquel 1865) is a prominent species of Salicaceae, 4–15 m tall and evergreen shrubs or small trees, which mainly distribute in forest margins, thickets on hills, plains and surrounding village at an altitude of 500–1100 m in the center, south and southwest of China and in India (rare), Japan and Korea (Yang and Sue 2007). It possesses high value for timber, medicinal, ornamental, and ecological purposes. But, to date, there is not full knowledge on the complete cp genome was measured for *X. racemosa*. The object of this work was to explore the intrinsic distinction in an effort to vindicate its taxonomic status in genus *Xylosma*. Also, to reveal the complete genome sequence of *X. racemosa* could play an important role in the protection, development and utilization of its resources. In this work, we characterized the complete cp genome sequence of *X. racemosa* (GeneBank accession number: MZ379835) based on Illumina pair-end sequencing data to provide a valuable complete cp genomic resource.

The fresh leaves of *X. racemosa* were collected from Nanjing Forestry University (**latitude** 32.0815 and **longitude** 118.8156) in Nanjing, Jiangsu, China. A specimen was deposited at the herbarium of Nanjing Forestry University (contact person: xuehongma@njfu.edu.cn) under the voucher number NF2021029. The sample of genome sequencing library was formed by PCR amplification, which was extracted on Illumina Novaseq platform by Nanjing Genepioneer Biotechnologies Inc. (Nanjing, China), and read long for PE150 sequencing.

The original reads was filtered by fastp (version 0.20.0), and the clean data was assembled into chloroplast genome with SPAdes (Bankevich et al. 2012). Then, the reference sequence (Genebank accession number: MN078135.1) was used for quality control after assembly. Finally, the assembled genome was annotated using CpGAVAS (Liu et al. 2012). Based on the Maximum Likelihood (ML), the phylogenetic tree was deduced by MAFFT (Rozewicki et al. 2019) and IQ-Tree (Gao et al. 2018).

The complete chloroplast genome sequence of *X. racemosa* was 157,262 bp in length, and contained a pair of inverted repeat (IRa and IRb) regions of 27,578 bp, which were separated by a large single copy (LSC) region of 84,289 bp, and a small single copy (SSC) region of 17,817 bp. A total of 131 genes are encoded, including 86 protein-coding genes (77 CDS species), 37 tRNA genes (30 tRNA species) and 8 rRNA genes (4 rRNA species). Most of genes occurred in a single copy; however, 9 protein-coding genes (*ndhB, rpl2, rpl23, rps12, rps19, rps7, ycf1, ycf15* and *ycf2*), 7 tRNA genes (*trnA-UGC, trnI-CAU, trnI-GAU, trnL-CAA, trnN-GUU, trnR-ACG* and *trnV-GAC*), and 4 distinct rRNA genes (*4.5S, 5S, 16S,* and *23S*) are duplicated. A total of 9 protein-coding genes (*atpF, ndhA, ndhB, petB, petD, rpl16, rpl2, rpoC1* and *rps12*) contained 1 intron while the other 2 genes (*clpP, ycf3*) had 2 introns each. The overall GC content of *X. racemosa* genome is 36.74%, and the corresponding values in LSC, SSC, and IR regions are 34.62, 30.38, and 42.04%, respectively.

To reveal the phylogenetic evolution of *X. racemosa*, we constructed a ML phylogenetic tree based on 16 cp genomes from Salicaceae and 3 cp genomes as outgroups from 2 taxa (Achariaceae, Malpighiaceae). We found that *X. racemosa* was clustered with other families of Salicaceae with 100% boot-strap values (Figure 1). In addition, *X. racemosa* was highly supported to be a sister species to *Xylosma congesta* in Salicaceae.

**Figure 1.**
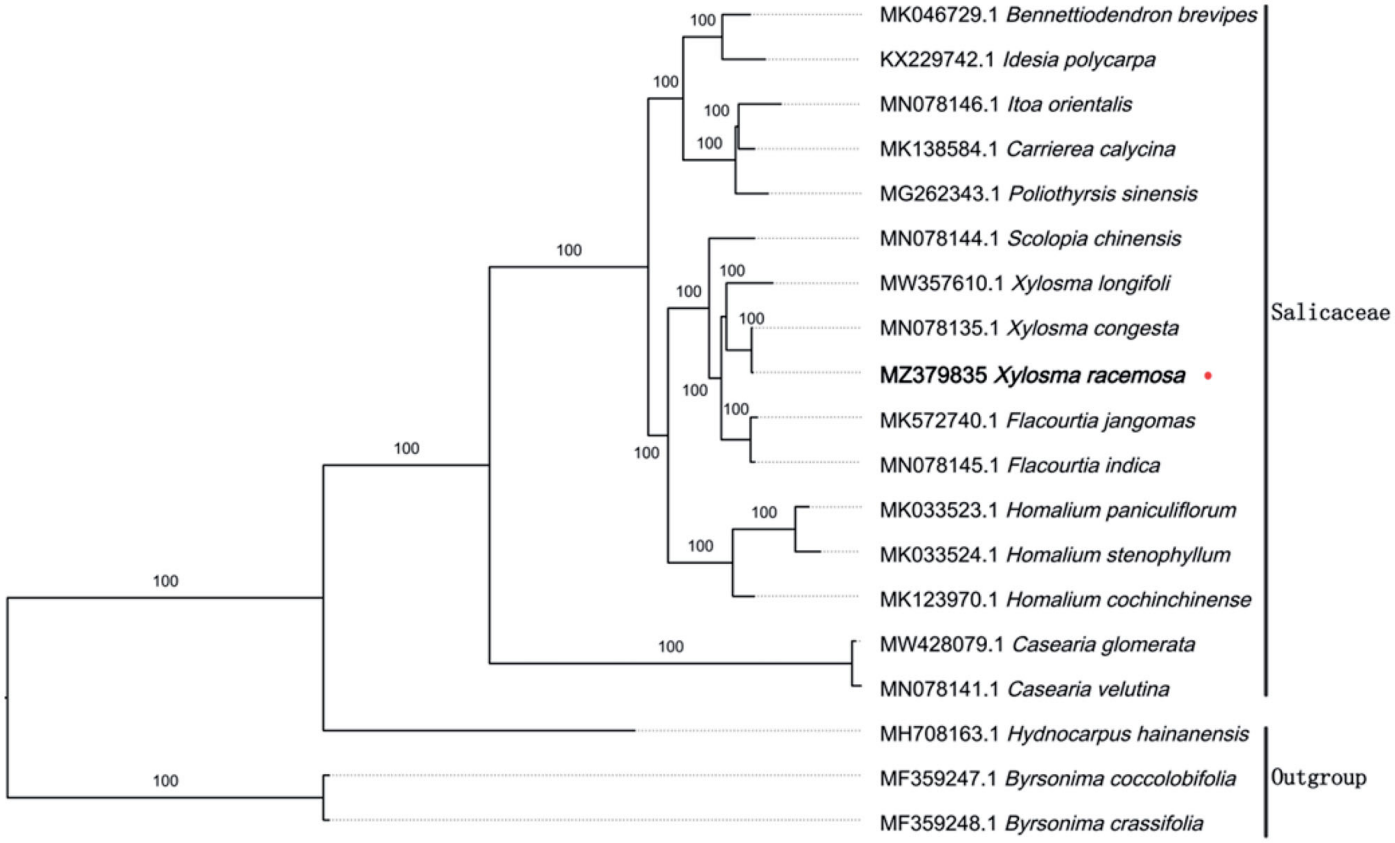
Maximum likehood tree showing the relationship among *Xylosma racemosa* and representative species within Salicaceae, based on whole chloroplast genome sequences, with 2 taxa from Malpighiales as outgroup. The bootstrap supports the values shown at the branches.

## Data Availability

The genome sequence data that support the findings of this study are openly available in GenBank of NCBI at [https://www.ncbi.nlm.nih.gov] (https://www.ncbi.nlm.nih.gov/) under the accession no. MZ379835. The associated BioProject, SRA, and Bio-Sample numbers are PRJNA739511, SRR14876284, and SAMN19790829, respectively.
